# Evaluation of a Community-Based Chronic Kidney Disease Screening Program at a Senior Living Community in Blacksburg, Virginia

**DOI:** 10.7759/cureus.101303

**Published:** 2026-01-11

**Authors:** Tobias K Fuchs, Ashley Funkhouser, Elizabeth Geddes, Youssef Moustafa, Michael DeGrassi, Waleed Iftikhar, Patrick Buchanan, Dawson Downing, Jessica Nicholson, Bernard Kadio

**Affiliations:** 1 Clinical Sciences, Edward Via College of Osteopathic Medicine, Blacksburg, USA; 2 Epidemiology and Public Health, Edward Via College of Osteopathic Medicine, Blacksburg, USA

**Keywords:** chronic kidney disease (ckd), community-based screening, geriatrics, nephrology, program evaluation, public health, survey study

## Abstract

Introduction: Chronic kidney disease (CKD) is often asymptomatic until advanced stages, predominantly affecting adults aged ≥65 years. The U.S. Preventive Services Task Force (USPSTF) currently states that there is insufficient evidence to perform routine screening for CKD in those who do not display symptoms. Without proper guidelines to routinely perform these tests, free screenings are scarce throughout the United States, but are offered by certain organizations such as the Kidney Disease Screening and Awareness Program (KDSAP). In October 2024, the Virginia Tech Chapter of KDSAP performed a free CKD screening at Warm Hearth Village (WHV) retirement home in Blacksburg, Virginia, an ideal population to catch early progression of this disease. This study aimed to understand if this screening increased awareness, convenience/satisfaction, follow-up adherence, and recommendations for program improvement.

Methods: A cross-sectional, anonymous survey was distributed to 36 residents who participated one year after the screening. The option of an electronic or paper copy of our survey was provided by the WHV staff and collected upon completion. No reminder or follow-up contact regarding surveys was provided.

Results: Eleven out of 36 participants responded to the survey. Results showed that the program was convenient or very convenient (72.8%), the program improved understanding of kidney health (81.8%), explanations varied in clarity (45.5% completely clear, 45.5% somewhat clear, 9% not clear), and the program left participants satisfied or very satisfied (63.6%), with strong interest in future screenings (81.8%). Every responder agreed with expansion to other communities (100%). Suggested areas for improvement include time and availability, wait times, explanation of results, and follow-up communication.

Conclusion: Community-based CKD screenings appear feasible and well-received, but improved scheduling, staffing, education, and explanation of results may enhance program effectiveness.

## Introduction

Chronic kidney disease (CKD) is a silent, slowly progressive disease that results in the gradual loss of nephrons and ultimately leads to kidney dysfunction [1]. Due to the number of years it takes to accumulate the decline in function, CKD is more common among adults aged 65 years and older [2,3].

As of yet, there is no screening recommendation provided by the U.S. Preventive Services Task Force (USPSTF) regarding CKD to detect this condition in its earlier stages [4]. The American College of Physicians has recommended against screening for CKD in asymptomatic adults who do not have any known risk factors [5]. Routine CKD screening is not recommended for individuals who are asymptomatic with no known risk factors due to insufficient evidence of benefit in low-risk populations; however, targeted screening is recommended for those who do show risk factors such as older age, hypertension, diabetes, or family history of kidney disease.

Due to the lack of recommendations, there are a limited number of programs nationwide that provide free screening opportunities through evaluation of blood testing, urinalysis, blood pressure measurement, and gauging lifestyle factors [6]. Although these tests are routinely available in clinical settings, barriers such as cost, access, scheduling, and limited engagement with primary care may reduce uptake, particularly among older adults. KDSAP is an organization that contains multiple student-led chapters at various universities across the United States, offering free CKD screenings not only for the community but also to increase interest in students to become future nephrologists [7-9]. Community-based programs such as KDSAP operate with an educational and risk-awareness focus, providing voluntary screening and referral rather than population-wide screening.

While these programs can detect abnormal values to provide referrals to physicians, there remains limited evidence on how effective these screening programs are, how they are perceived by participants, or if they can improve in any capacity, particularly among older adults [4,6]. This study sought to determine how effective a kidney screening at Warm Hearth Village (WHV), a retirement home located in Blacksburg, Virginia, was in October of 2024, provided through KDSAP, a Virginia Tech-affiliated organization. Effectiveness was determined through a one-year follow-up survey that asked a multitude of questions regarding satisfaction, convenience, result clarity, and adherence to referral/follow-up. The objective of this program evaluation was to describe participant-reported perceptions of accessibility, satisfaction, clarity of result communication, and adherence to referral and follow-up recommendations using a one-year post-screening survey. In this evaluation, effectiveness refers to participant-perceived benefit and self-reported follow-through rather than diagnostic accuracy. Ultimately, the findings from this program evaluation may help clarify participant-perceived effectiveness and inform improvements to future free, one-day kidney screening programs.

## Materials and methods

This study was conducted as a descriptive, cross-sectional program evaluation using a post-screening follow-up survey to assess participant-reported perceptions of a community-based CKD screening. In October of 2024, the KDSAP organization set up and ran a free kidney screening initiative at the WHV retirement home. Undergraduate students in the organization coordinated with the WHV staff for approval and assistance in running the kidney screening event. WHV community members were given the opportunity to register for the free screening. Upon arrival, they were instructed to fill out paperwork. Education was provided regarding the purpose of the screening, what lifestyle factors contribute to disease progression, and recommendations on how to prevent disease occurrence. Then measurements were obtained for every participant, including their body mass index, urinalysis, blood pressure reading, and blood glucose levels. After all the data were collected for each participant, a physician consultation was provided, which could include referral for follow-up [8,10]. Participants were informed that the screening was voluntary, educational in nature, and not a substitute for routine clinical care.

Thirty-six WHV residents participated in the screening. Through the WHV staff, a cross-sectional anonymous follow-up survey was administered to all 36 participants one year following the event, either via an email that contained a survey link or via a paper copy. Both forms of electronic and paper response were offered to increase response rate, as paper may be preferred over online surveys in older populations [11]. All individuals who attended the screening were eligible to complete the follow-up survey, and no additional inclusion or exclusion criteria were applied. WHV staff collected 11 responses, which were provided to the research team. The survey contained 18 questions and only collected de-identified information that could not be linked to participants (Appendix A). Survey completion time was about five to 10 minutes. Questions were directed towards participant demographics, convenience, education effectiveness, clarity of results, next steps, motivation, satisfaction, suggestions for improvement, and if further screenings are recommended. Response formats included Likert scale, yes/no, multiple-choice, and free-response.

Due to the small sample size (11/36), descriptive analysis was performed, summarizing responses as counts or percentages. No inferential statistics were performed.

The Edward Via College of Osteopathic Medicine Institutional Review Board (Blacksburg, Virginia) reviewed this program evaluation and determined exemption from full review (Institutional Review Board (IRB) exemption number: 2025-058). Participation was voluntary, and completion of the anonymous survey was treated as implied consent. This project was IRB approved on September 26, 2025.

## Results

Eleven of 36 participants who attended the screening responded one year following the event. All respondents recommended expanding the screening to other communities to detect early cases of kidney decline/dysfunction and thought the screening was easily accessible (11/11, 100%). Nine of 11 respondents stated the educational aspect of the screening improved their understanding of kidney health and that they would participate in a future kidney screening (81.8%). Eight out of 11 respondents stated that the program was convenient (72.7%). Seven out of 11 respondents stated that they were satisfied with the screening (63.6%). Only five out of 11 respondents stated that the explanation of the results from the physician was clear (45.5%). Detailed response counts and percentages are presented in Table 1. Importantly, one individual was noted to have abnormal values and was referred to follow-up care and attended the follow-up visit. When asked which part of the program was most useful, participants most frequently cited the free testing and educational components. When given a chance for free response in regard to how the program can be improved, multiple respondents stated that shortening wait times between stations would be preferred and that one physician was not enough for consultation. One respondent reported leaving before receiving their results. There were concerns that the physician could have spent more time explaining the results in lay terms so that participants could understand more clearly. Suggestions for providing some form of follow-up communication regarding results and next steps were made.

**Table 1:** Summary of Participant-Reported Outcomes Following the Warm Hearth Village Chronic Kidney Disease Screening (n = 11) Numerical values and percentages are based on 11 participant responses. Percentage totals may not equal 100 due to rounding.

Outcome domain	Response definition	n (%)
Program accessibility	Screening reported as easily accessible	11 (100)
Program expansion	Recommended expansion to other communities	11 (100)
Educational benefit	Improved understanding of kidney health	9 (81.8)
Future participation	Would participate in a future kidney screening	9 (81.8)
Program convenience	Rated the program as convenient	8 (72.7)
Overall satisfaction	Reported being satisfied with the screening	7 (63.6)
Clarity of result explanations	Reported explanations were clear	5 (45.5)
Follow-up attendance	Attended follow-up visit after referral	1 (9.1)

Detailed item-level response distributions for all survey questions are provided in Appendix B.

## Discussion

This program evaluation showed that the KDSAP kidney screening was accessible, convenient, and satisfactory for most. A one-year follow-up interval was intentionally selected to assess longer-term retention of educational content and to determine whether recommended clinical follow-up actions had been completed over a full annual cycle. Participants stated they would consider attending future screenings and would like to see these screenings expand beyond just their own community. Most participants had an increased understanding of kidney health and what lifestyle factors can contribute to or prevent CKD.

Previous studies have focused on evaluating whether CKD screenings can detect kidney disease early on, showing that identification of undiagnosed CKD can occur in a large number of individuals through screening [12]. Other studies have focused on the cost-effectiveness of screenings in the general population and those with specific conditions like diabetes or hypertension [13,14]. Findings noted that CKD screenings were cost-effective for those with diabetes or hypertension but also for older adults [15]. In contrast, limited evidence exists for how these screenings are perceived among the participants in at-risk groups or how the structure and delivery can be improved. Providing recommendations for the logistical and educational aspects of these screenings can increase positive perceptions in the future and promote behaviors that can slow CKD progression [16].

A prior study evaluated a CKD screening by examining how patients perceived the screening, satisfaction, and service quality [17]. Our study and Lin et al. [17] both found that participant satisfaction was high, low perception scores were indicated by poor responsiveness and communication, and participants strongly recommended the screening to others. Lin et al. did find that perception scores were lower among older adults. Key differences in their study include that the surveys were mainly completed by the general community with a mean age of 45 years, with surveys completed on-site or shortly after screening, whereas our evaluation focused on a retirement community with a mean age of 65 years and older, with surveys being completed one year post event. Overall, both our study and Lin et al. [17] found that while screenings are well-received, there is room for improvement in service delivery.

Although our study identified only one individual who was referred for follow-up care and attended their appointment, this observation is consistent with prior findings that showed those with declining kidney function have higher follow-up rates [18].

Based on our findings, future screenings can increase efficiency and effectiveness by scheduling time slots for arrival, increasing the number of volunteers per station, and increasing the number of physicians for consultations to shorten wait times. During consultation, it would be preferred that physicians take more time with each evaluation and be prepared to use simplified terminology to assure participant understanding. Before exiting the screening, participants could be asked what method of communication they prefer, and results could be sent via the preferred route with follow-up steps (email, text, or printed) or reminders of general education on lifestyle factors. If a referral is indicated, offering on-site appointments for follow-up and checking in one week after the appointment could increase adherence to follow-up and provide a reliable linkage to care.

Interpretation of these findings is limited by the high nonresponse rate, which restricts generalizability and may bias participant-reported satisfaction toward more favorable perceptions. Various limitations have been noted during this evaluation, including a small sample size with a sub-optimal response rate (31%). Reduced response rate could be due to administering the surveys one year following the screening, which could have led to loss of recall, loss of confidence in details, or a general change in their health/residence that restricted their participation [19]. Recall bias could also be a factor, as details could be incorrectly remembered one year following the event. Surveys were administered to older adults, who are known to have a lower response rate to electronic surveys [11]. Distribution and collection of surveys occurred through the WHV facility staff, with only one attempt to reach out to screening participants for response. No reminders or follow-up attempts were made to complete the survey. Responses were self-reported, with the possibility of response bias being present because not all participants in the screening responded. Individuals with more favorable perceptions may have been more likely to respond, potentially biasing satisfaction outcomes toward more positive results. Because surveys were distributed and collected by WHV staff and only de-identified responses were provided to the research team, demographic data were not available for non-responders, limiting the ability to assess differences between respondents and those who did not respond. This screening occurred at one location in Blacksburg, Virginia, through one organization (KDSAP). Future evaluations should seek to increase the number of participants/respondents and offer surveys onsite or soon after the screening event. Evaluation of programs should be sought through multiple organizations that offer free CKD screenings, as the workflow process may differ between programs. Evaluations should be expanded beyond Blacksburg, Virginia, to get a better understanding of the true effectiveness of these CKD screenings nationwide.

## Conclusions

This program evaluation provides descriptive insight into participant-reported experiences following a community-based CKD screening among respondents aged 65 and older. The WHV CKD screening that took place in October 2024 through the KDSAP organization was positively received among participants. Many expressed that these screenings are beneficial and should be expanded to other communities. Satisfaction, accessibility, and convenience scores were consistently rated as being high; however, certain areas for improvement were highlighted to enhance the effectiveness of future screenings. Improvements include better organization and preparation by increasing staffing to shorten wait times, physicians spending more time with each participant, and using simple terminology to increase clarity of results. Follow-up with a copy of the results should be provided through the participants' preferred route of communication. Providing linkage of care through onsite referral appointments should also be considered to further improve the effectiveness of future CKD screenings.

## Appendices

Appendix A 

**Table 2:** Post-screening Follow-Up Survey Instrument

Question No.	Survey question	Response options
1	Age	Free response
2	Gender	Male / Female / Other
3	Occupation	Free response
4	Education level	None / Primary / Secondary / Tertiary
5	Do you have a family history of kidney disease?	Yes / No
6	How did you hear about this kidney screening program?	Health worker / Community campaign / Media / Friends or family / Other
7	Was the screening location easily accessible to you?	Yes / No
8	How would you rate the convenience of the program (time, location, cost)?	Very convenient / Convenient / Neutral / Inconvenient / Very inconvenient
9	Did the screening program help you understand your kidney health better?	Yes, very much / Somewhat / Not really / Not at all
10	Did you receive clear explanations about your screening results?	Yes, completely / Somewhat / No
11	Has this program motivated you to take steps to protect your kidney health?	Likert scale (1 = Strongly disagree, 5 = Strongly agree)
12	Do you feel the screening program will help in the early detection of kidney problems in the community?	Yes / No / Not sure
13	Overall, how satisfied are you with the program?	Very satisfied / Satisfied / Neutral / Dissatisfied / Very dissatisfied
14	What part of the program did you find most useful?	Free testing / Health education / Early detection / Counseling / Other
15	What improvements would you suggest for future kidney screening programs?	Free response
16	Would you be willing to participate in regular kidney screening in the future?	Yes / No
17	If you were referred for follow-up care, did you attend?	Yes / No / Not applicable
18	Do you think this program should be expanded to other communities?	Yes / No

Appendix B 

**Table 3:** Item-Level Response Distributions for the Post-screening Survey Percentages are based on 11 respondents and may not total 100% due to rounding. Free-response items are summarized qualitatively and are not presented as quantitative distributions.

Question No.	Survey question (exact wording)	Response distribution
Q1	Age	Mean 75.6 years (SD 8.8); range 59–88
Q2	Gender	Female 7 (63.6%); Male 4 (36.4%); Other 0 (0%)
Q3	Occupation	Retired 6 (54.5%); Retired teacher 1 (9.1%); Retired professor 1 (9.1%); Retired nurse 1 (9.1%); Retired physician 1 (9.1%); Unemployed 1 (9.1%)
Q4	Education level	Tertiary 8 (72.7%); Secondary 2 (18.2%); Primary 1 (9.1%); None 0 (0%)
Q5	Do you have a family history of kidney disease?	Yes 1 (9.1%); No 10 (90.9%)
Q6	How did you hear about this kidney screening program?	Community campaign 8 (72.7%); Warm Hearth 1 (9.1%); WHV program 1 (9.1%); Email 1 (9.1%); Health worker 0 (0%); Media 0 (0%); Friends or family 0 (0%); Other 0 (0%)
Q7	Was the screening location easily accessible to you?	Yes 11 (100%); No 0 (0%)
Q8	How would you rate the convenience of the program (time, location, cost)?	Very convenient 4 (36.4%); Convenient 4 (36.4%); Neutral 3 (27.3%); Inconvenient 0 (0%); Very inconvenient 0 (0%)
Q9	Did the screening program help you understand your kidney health better?	Yes, very much 3 (27.3%); Somewhat 6 (54.5%); Not really 1 (9.1%); Not at all 1 (9.1%)
Q10	Did you receive clear explanations about your screening results?	Yes, completely 5 (45.5%); Somewhat 5 (45.5%); No 1 (9.1%)
Q11	Has this program motivated you to take steps to protect your kidney health?	Strongly disagree (1): 0 (0%); Disagree (2): 1 (9.1%); Neutral (3): 4 (36.4%); Agree (4): 5 (45.5%); Strongly agree (5): 1 (9.1%)
Q12	Do you feel the screening program will help in the early detection of kidney problems in the community?	Yes 7 (63.6%); Not sure 4 (36.4%); No 0 (0%)
Q13	Overall, how satisfied are you with the program?	Very satisfied 3 (27.3%); Satisfied 4 (36.4%); Neutral 4 (36.4%); Dissatisfied 0 (0%); Very dissatisfied 0 (0%)
Q14	What part of the program did you find most useful?	Free testing 5 (45.5%); Early detection 2 (18.2%); Counseling 2 (18.2%); Health education 1 (9.1%); Other 1 (9.1%)
Q15	What improvements would you suggest for future kidney screening programs?	Free-response; summarized qualitatively in Results and Discussion
Q16	Would you be willing to participate in regular kidney screening in the future?	Yes 9 (81.8%); No 2 (18.2%)
Q17	If you were referred for follow-up care, did you attend?	Yes 1 (9.1%); Not applicable 10 (90.9%)
Q18	Do you think this program should be expanded to other communities?	Yes 11 (100%); No 0 (0%)
